# *STAT3* regulates NK and NKT cell differentiation through C-X3-C motif chemokine receptor 1  (CX3CR1) in hyper-IgE syndrome

**DOI:** 10.1186/s43556-025-00323-1

**Published:** 2025-11-10

**Authors:** Ju Liu, Jingzhi Yang, Jianing Tang, Hongxia Tang, Xin Dai, Peiyao Jin, Yanmei Huang, Zhenzhen Li, Ziyin Zhang, Xiaohuan Guo, Martin Bitzan, Xiaoling Yin, Chaohong Liu

**Affiliations:** 1 Department of Pathogen Biology, School of Basic Medicine, Tongji Medical College and State Key Laboratory for Diagnosis and Treatment of Severe Zoonotic Infectious Diseases, Huazhong University of Science and Technology, Wuhan, 430030 Hubei China; 2 Department of Pediatrics, Tongji Medical College of Huazhong University of Science and Technology, Wuhan, 430030 Hubei China; 3 Department of Orthopedics, Qilu Hospital of Shandong University, Jinan, Shandong China; 4 Wuhan Children’s Hospital, Tongji Medical College, Huazhong University of Science & Technology, Wuhan, China; 5 Institute for Immunology, Tsinghua University, Beijing, China; 6 Department of Basic Medical Sciences, School of Medicine, Tsinghua University, Beijing, China; 7 Beijing Key Lab for Immunological Research On Chronic Diseases, Tsinghua University, Beijing, China; 8 Central People’s Hospital of Zhanjiang, Zhanjiang, China; 9 Department of Immunology, School of Medicine, Yangtze University, Jingzhou, China; 10 Kidney Centre of Excellence, Al Jalila Children’s Hospital, Dubai, United Arab Emirates

**Keywords:** STAT3 mutation, Hyper-IgE syndrome, C-X3-C motif chemokine receptor 1, NK cells

## Abstract

Mutations in the signal transducer and activator of transcription 3 (*STAT3*) gene are strongly associated with Hyper-IgE Syndrome (HIES), a rare immunodeficiency disorder characterized by elevated levels of IgE and recurrent infections. The molecular mechanisms of how STAT3 dysfunction contributes to the pathophysiology of HIES are complex and not fully elucidated, especially in natural killer (NK) cells, which are crucial for the immune response against infections and malignancies. Employing single-cell sequencing and flow cytometry, we investigated the effects of *STAT3* mutations on immune cell development, differentiation, and function. Our findings revealed an increased population of CX3CR1^+^CD57^+^ NK and NKT cells, suggesting their terminal differentiation and functional exhaustion. The trend of Th2 cell differentiation was identified in patients with *STAT3* mutations and in STAT3 conditional knockout (CKO) mice. CUT&Tag analysis on CD4^+^ T cells from carriers of the *STAT3* intron22 (2144 + 1G > A) mutation revealed enhanced binding of the variant STAT3 to the transcription start site of *IL-4*, which provides an explanation for the elevated peripheral IgE levels observed in these *STAT3* mutation patients. This study enhances our understanding of how *STAT3* mutations drive immunological dysregulation in HIES. The identified changes in immunological signature and transcriptional mechanisms offer new insights into therapeutic targets for HIES.

**Supplementary Information:**

The online version contains supplementary material available at 10.1186/s43556-025-00323-1.

## Introduction

As a member of the signal transducers and activators of transcription (STAT) family, STAT3 plays a pivotal role in signal transduction, particularly in response to cytokines such as interleukin-6 (IL-6). The activation of STAT3 is a key event in the IL-6-induced acute-phase response [1, 2]. Notably, the hyperactivation of the IL-6/JAK/STAT3 pathway is generally associated with a poor clinical prognosis, possibly due to its influence on inflammation and immune cell behavior [3, 4]. STAT3 is organized into six functionally conserved domains: the amino-terminal domain (NTD), the coiled-coil domain (CCD), the DNA-binding domain (DBD), the linker domain (LD), the Src homology 2 domain (SH2), and the carboxyl-terminal transactivation domain (TAD) [5, 6]. Each of these domains contributes to its signaling functions [7] that are involved in myriad biological processes. De novo mutations in *STAT3* are responsible for most cases of Autosomal Dominant Hyper-IgE Syndrome (AD-HIES) [8–10], which are primarily caused by loss-of-function (LOF) mutations. AD-HIES, also known as Job’s syndrome, is characterized by elevated serum IgE, recurrent infections, and allergic symptoms [11–13]. Despite the established link between *STAT3* mutations and HIES, the detailed molecular and cellular mechanisms leading to the clinical manifestations of the disease remain unclear.

Immune dysregulation in HIES is due to both STAT3 loss- and gain-of-function mutations. CD4^+^ T cells play a crucial role in orchestrating both innate and adaptive immunity to defend against infections, but they also contribute to the pathogenesis of allergies and autoimmune diseases [14]. Th2 cells promote B cell proliferation and IgE production, primarily through the secretion of IL-4, IL-5, and IL-13. Among these, IL-4 is the principal driver of immunoglobulin class switching to IgE [15, 16]. Indeed, previous research has demonstrated an inverse correlation between STAT3 transcriptional activity and serum IgE concentration [17]. While the IL-6/STAT3 signaling pathway has been implicated in regulating IgE levels, the precise mechanisms by which *STAT3* LOF mutations impact T cell homeostasis and lead to elevated serum IgE still require further investigation.

NK and NKT cells are innate immune cells that are some of the first effectors recruited to sites of inflammation [18]. Human NK cells can polarize into two distinct functional subsets, termed NK1 and NK2 cells. Evidence indicates that the NK1 subset suppresses IgE production [19, 20]. In the adaptive immune system, CX3CR1, a chemokine receptor for fractalkine (CX3CL1) that mediates leukocyte adhesion and migration, has been established as a hallmark of functionally mature and cytotoxic effector T cells [21, 22]. In the innate immune system, CX3CR1 guides NK cell migration to inflammatory sites [19] and is often associated with enhanced cytotoxicity and migratory capacity [23, 24]. The chemokine CX3CL1 and its receptor CX3CR1 constitute a critical signaling axis, expressed across a wide range of immune and non-immune cells. This interaction is integral to host defense, notably by facilitating the “patrolling” behavior of immune cells [25]. However, other research has demonstrated that under certain pathological conditions, CX3CR1^+^ NK cells exhibit impaired functions, suggesting that the relationship between CX3CR1 and NK cells merits further investigation [26].

In this study, we address these gaps by investigating patients with STAT3-deficient HIES. We discovered a significant expansion of terminally differentiated CX3CR1^+^CD57^+^ NK and NKT cells, and subsequently uncovered a previously unrecognized regulatory axis where STAT3 directly suppresses CX3CR1 expression. Concurrently, we elucidated a mechanism for the hallmark hyper-IgE phenotype, demonstrating that loss of STAT3 function leads to upregulated IL-4 production and an augmented Th2 response. Together, our findings not only provide novel insights into the pathophysiology of HIES but also reveal the broader role of STAT3 in maintaining the homeostasis of cytotoxic lymphocyte populations.

## Results

### Case history of *STAT3* Intron22 (2144 + 1G > A) mutation

**Fig. 1:**
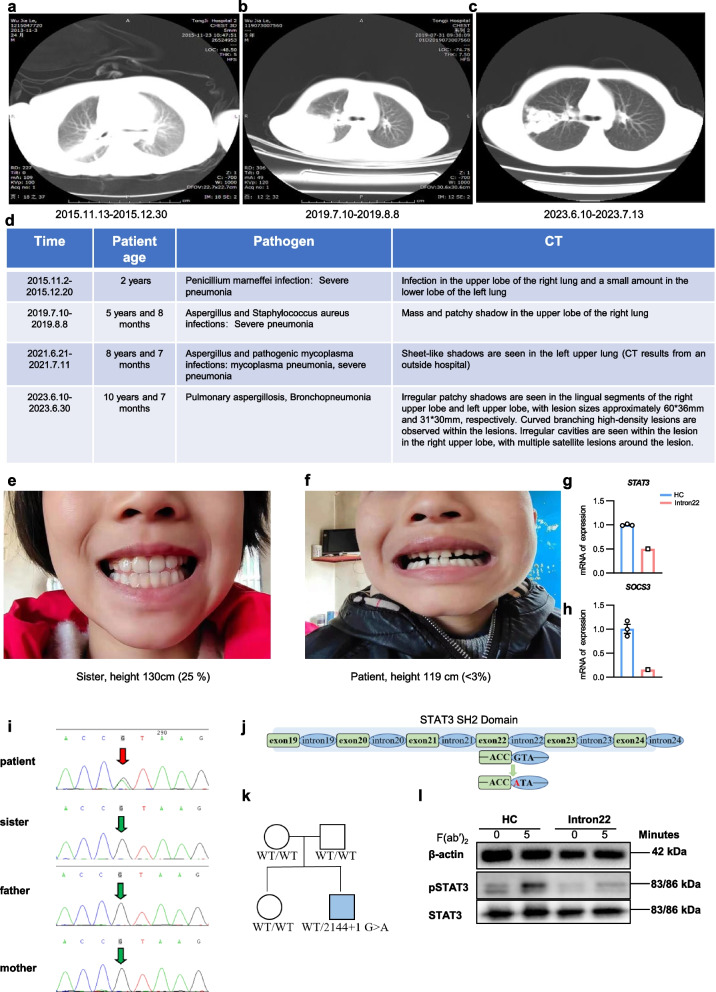
Clinical manifestations and identification of children with *STAT3* intron22 mutation. **a**-**c** Representative chest computed tomography (CT) scans of the patient during three separate hospitalizations for severe pulmonary infections, showing progressive lung damage. Images correspond to infections with *Penicillium marneffei* (2015) (**a**), *Aspergillus* and *Staphylococcus aureus* (2019) (**b**), and *Pulmonary aspergillosis* and *bronchopneumonia* (2023) (**c**). **d** A summary table of the patient’s history of severe infections, including identified pathogens and corresponding CT findings. **e**–**f** Clinical photographs comparing the facial and dental features of the patient’s healthy sister (**e**) with the patient (**f**) at 7.5 years old. The patient exhibits characteristic features of HIES, including a coarse facial appearance, retention of primary teeth, and severe growth retardation (height 119 cm, < 3rd percentile) compared to his sister (height 130 cm, 25th percentile). **g**-**h** qPCR analysis of the mRNA levels of *STAT3* and *SOCS3* in PBMCs from the patient and healthy controls (HCs). Data are presented as mean ± SEM. (HCs, n=3). **i** Sanger sequencing chromatograms confirming a heterozygous c.2144 + 1G > A mutation in intron22 of the *STAT3* gene in the patient, which was absent in his unaffected sister and parents. The red arrow indicates the mutation site. **j** Schematic diagram of the *STAT3* SH2 domain, illustrating the location of the splicing mutation at the + 1 position of intron22. **k** Pedigrees of the families with heterozygous *STAT3* intron22 mutation. **l** Immunoblot analysis of total STAT3 and phosphorylated-STAT3 (pSTAT3) in PBMCs from the patient and HC. Cells were either unstimulated (0 min) or stimulated with F(ab’)₂ for 5 min. β-actin serves as a loading control

The index patient, a 10-year-old boy, was the second child of nonconsanguineous parents of Chinese origin. He has a 12-year-old sister without apparent health concerns. At the age of 2 years, the patient experienced a severe pulmonary infection caused by Penicillium marneffei (PM), a pathogenic fungus affecting patients with HIV infection. The chest CT scan showed infection in mid portions of both lungs (Fig. 1a). He progressed to respiratory and heart failure. Serum tests revealed an elevated IgE level of 2626.75 IU/mL (normal 0–90 IU/mL). The patient was hospitalized and treated with amphotericin B, and control over the infection was achieved after 37 days. At the age of 5.7 years, the patient developed another episode of severe pneumonia caused by Aspergillus sp. and methicillin-resistant *Staphylococcus aureus*, identified in the bronchial lavage specimen. A chest CT scan showed a mass and a patchy shadow in the upper lobe of the right lung (Fig. 1b). The patient was treated with voriconazole and teicoplanin for 28 days. At the ages of 8 and 10 years, the patient experienced severe coughing and fever, leading to hospitalizations where severe pneumonia was diagnosed based on CT scans (Fig. 1c-d). Notably, the patient’s hospitalization duration after each infection exceeded 20 days, substantially longer than the typical 7–14 day hospital stay for children with severe infections (Fig. 1d). During the most recent hospitalization, the patient’s serum IgE level was 3200 IU/mL. His skeletal development was delayed, and he still had his primary teeth at his last visit at the age of 10 years. His teeth were small and spread abnormally wide. His growth was below the 3rd percentile for children of the same age (Fig. 1e-f). Also, the patient had eczematous skin and multiple food allergies, such as milk, eggs, beef, protein, fish, and shrimp. Serum IgE levels remained elevated between infections. Clinical and laboratory findings suggested HIES and a mutation in the *STAT3* gene. This suspicion was confirmed by genetic sequencing, which identified a heterozygous mutation of 2144 + 1G > A within *STAT3* intron22 (Fig. 1i)*.* Further genetic sequencing of the patient’s family members confirmed that the mutation arose de novo (Fig. 1i-k). The patient did not receive treatments targeting IL-4, IgE, or the STAT3 pathway. Peripheral blood mononuclear cells (PBMCs) from the patient showed comparable levels of total STAT3 protein across conditions; however, upon stimulation with biotinylated F(ab’)_2_ anti-human Ig(M + G), there was a notable reduction in tyrosine-phosphorylated STAT3 (pSTAT3) (Fig. 1l). We further assessed the mRNA levels of *STAT3* and *SOCS3*, a downstream regulatory gene of the STAT3 pathway, and observed a significant decrease in their expression (Fig. 1g-h), which suggests an impairment in the activation of the STAT3 signaling pathway.

### Immune cell profiling of PBMCs using single-cell RNA sequencing

PBMCs from the patient with intron22 mutation and age-matched healthy children were collected for scRNA-seq. Transcriptional profiling was performed using droplet-based scRNA-seq (10 × genomics) analysis after sequencing data were processed by the CellRanger program. A total of 3770 single cells were obtained after quality control procedures.

**Fig. 2:**
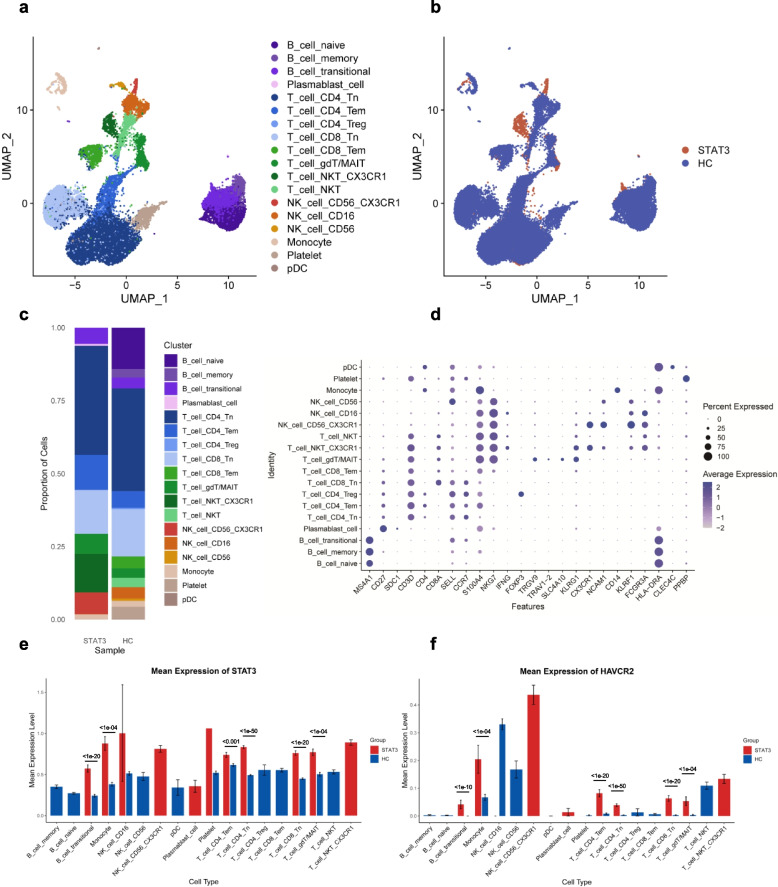
scRNA-seq identifies novel CX3CR1^+^ NK and NKT cell clusters in PBMCs from a patient with a *STAT3* intron22 mutation. **a**-**b** Integration analysis results of *STAT3* intron22 (2144 + 1 G > A) mutation patient and three HCs, showing the UMAP formation of 18 clusters (**a**) and status (**b**). **c** The relative abundance of immune cells in the PBMCs of each cluster. **d** Dot plots showing the expression of selected canonical cell markers in the 18 clusters. Deeper colors represent higher expressions, and larger dots represent larger populations of immune cells that express the cell markers. **e**–**f** The expression level of STAT3 and TIM3 (HAVCR2) on each immune cells from scRNA-seq data

After unsupervised hierarchical clustering, 6 major cell types were identified, including B cells, T cells, monocytes, NK cells, pDCs, and platelets. Among them, B cells comprised 4 subtypes (naïve, memory, transitional B cells, and plasmablasts), T cells exhibited 8 subtypes: CD4^+^ naïve T (CD4^+^ Tn), CD8^+^ naïve T (CD8^+^ Tn), CD4^+^ effector memory T (CD4^+^ TEM), CD8^+^ effector memory T (CD8^+^ TEM), CD4^+^ regulatory T (Treg), γδ T cells, mucosal-associated invariant T (gdT/MAIT) cells, natural killer T (NKT) cells, and CX3CR1^+^ NKT cells (NKT_CX3CR1). NK cells were categorized into three subtypes: CD56^+^ NK, CD16^+^ NK, and CD56^+^CX3CR1^+^ NK cells (Fig. 2a-d). The intron22 mutation sample also showed fewer naïve B cells, CD16^+^ NK cells, Tregs, and CD8^+^ TEM (Fig. 2c). The significant decrease in the proportion of B cells indicated a suppressed humoral immune response or impaired B cell maturation. Moreover, the decrease in B cell proportions alongside the increase in NK and NKT cell proportions suggested a shift towards innate immunity and potentially a state of immune dysregulation, which needed further investigation.

We further evaluated gene expression profiles in this patient with intron22 mutation. An intriguing observation was that the mRNA of *STAT3* exhibited a significant reduction in NKT and CD56^+^ NK cells, yet a notable increase was observed in CX3CR1^+^ NKT and CD56^+^CX3CR1^+^ NK cells, suggesting a potential association between STAT3 and CX3CR1 (Fig. 2e). A similar pattern of expression was also observed for T cell immunoglobulin mucin-3 (TIM3/HAVCR2) (Fig. 2f). Our qPCR results detected a decrease in the total *STAT3* mRNA content in the PBMCs of the mutation carrier (Fig. 1g), which may be attributed to an increased proportion of cells with low expression of *STAT3* mRNA.

Utilizing Gene Ontology (GO) and Kyoto Encyclopedia of Genes and Genomes (KEGG) analyses, we found that CX3CR1^+^ NK and NKT cells are characterized by high metabolic features (Fig. S1i-j). Pseudotime analysis revealed that PBMCs from the intron22 mutation patient displayed distinctly different pseudotime trajectories compared to normal samples. Notably, NK cells, T cells, and B cells in the mutant sample were all positioned at the late stage of pseudotime, as determined by the placement of naïve cells. This positioning indicates that the intron22 mutation influences the differentiation and activation of these immune cells, leading to an increased proportion of more mature and differentiated cells. Branched expression analysis of transcriptional changes in immune genes in lymphocytes and monocytes also demonstrated enrichment in pathways involved in cell differentiation and activation, such as signal regulation, macromolecule localization, and phosphorylation. Specifically, monocytes and NK cells showed an enrichment of regulation of the actin cytoskeleton, while T and B cells exhibited an enrichment of antigen processing and presentation (Fig. S1a-h). These findings were consistent with the pseudotime analysis, indicating a hyperactivated state of immune cells with intron22 mutation, which warranted further investigation.

### *STAT3* mutation leads to altered NK and NKT cell subsets with high CX3CR1 expression

**Fig. 3:**
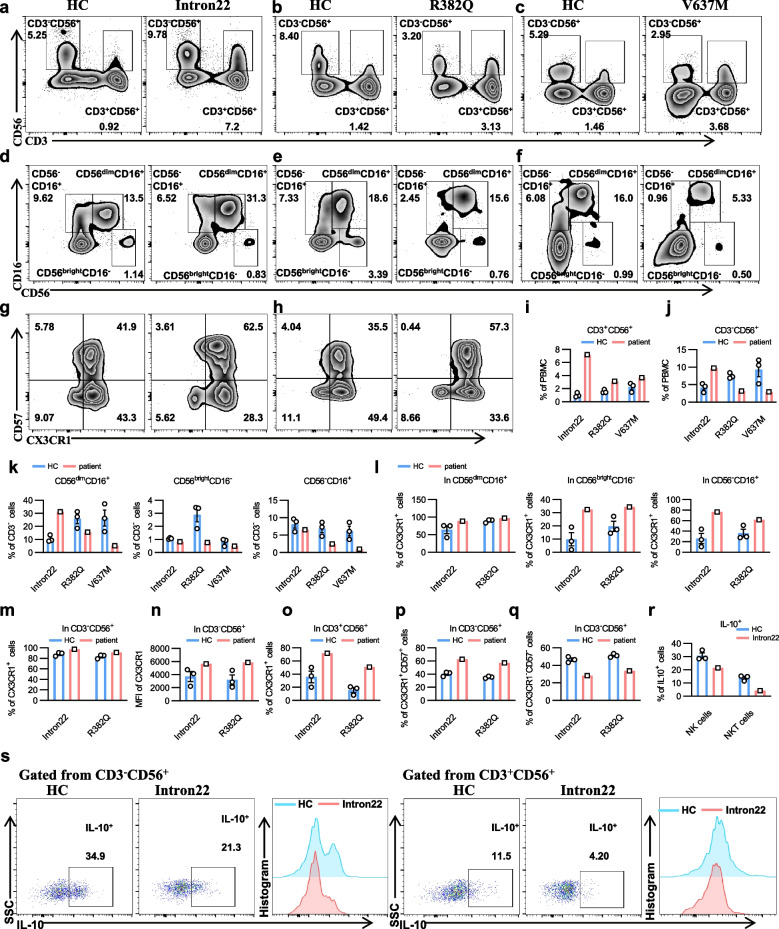
Flow cytometry analysis reveals the presence of CX3CR1^+^ NK and NKT cells in patients with *STAT3* mutations. **a**-**f** Flow cytometry (FCM) analysis of total NK (CD3^−^CD56^+^) and NKT cells (CD3^+^CD56^+^), as well as NK subpopulations (CD56^bright^CD16^−^, CD56^dim^CD16^+^, and CD56^−^CD16^+^) in PBMCs from three HCs and the *STAT3* mutant patients. Shown are representative dot plots. **g**-**h** FCM analysis of CX3CR1 and CD57 expression in CD3^−^CD56^+^ NK cell subsets in PBMCs from three HCs and *STAT3* mutant patients. Shown are representative dot plots. **i**-**k** Statistics of percentage (± SEM) of NKT (**i**), total NK (**j**) cells, CD56^bright^CD16^−^, CD56^dim^CD16^+^, and CD56^−^CD16^+^ NK cells from three HCs and the *STAT3* mutant patients. **l**-**o** Statistics of percentage (± SEM) of CX3CR1^+^CD56^bright^CD16^−^, CX3CR1^+^CD56^dim^CD16^+^, CX3CR1^+^CD56^−^CD16^+^ NK (**l**), CX3CR1^+^ NK cells (**m**), and CX3CR1^+^ NK cells (**o**) from three HCs and *STAT3* mutant patients. **p**-**q** Statistics of percentage (± SEM) of CX3CR1^+^CD57^+^ and CX3CR1^+^CD57^−^ cells in CD3^−^CD56^+^ NK cells from three HCs and *STAT3* mutant patients. **r**-**s** FCM analysis of IL-10^+^ cells in CD3^−^CD56^+^ NK and NKT cells from three HCs and *STAT3* mutant patients. Shown are representative dot plots (s). The percentage (± SEM) of IL-10^+^ cells was shown in (**r**)

Based on the single-cell analysis results, which revealed an elevated presence of CX3CR1^+^ NK and NKT cells in the intron22 mutant patient, we conducted further validation through flow cytometry analysis (Fig. S2a). We enrolled two additional patients with STAT3 mutations for validation: one with the R382Q mutation and another with the V637M mutation. NK and NKT cells were distinguished by CD3 and CD56. CD3^+^CD56^+^ cells were defined as NKT cells and CD3^−^CD56^+^ cells were defined as NK cells (Fig. 3a-c). Flow cytometry results showed that the proportion of NKT cells increased in all three patients (Fig. 3i), with the CD3^−^CD56^+^ NK cells being significantly decreased in R382Q and V637M mutation patients, but increased in the intron22 mutation patient (Fig. 3j). CD3^−^ NK cells are subdivided into three subpopulations based on cell surface expression of CD56 and CD16: CD56^−^CD16^+^, CD56^bright^CD16^−^, and CD56^dim^CD16^+^ (Fig. 3d-f). Results showed that the proportion of CD56^dim^CD16^+^ NK cells in the intron22 mutation sample was increased, while the CD56^−^CD16^+^ and CD56^bright^CD16^−^ subsets were decreased compared to HCs. In patients with R382Q and V637M mutation, the proportion of all three NK cell subsets were decreased (Fig. 3d-f, 3k). We further validated the effect of STAT3 on NK cell differentiation in a STAT3 CKO mouse model. STAT3 knockout significantly reduced the proportion of NK cells (Fig. S2b-d). By conducting flow cytometry, we further found that the proportion of CX3CR1^+^ cells and the mean fluorescence intensity (MFI) of CX3CR1 in CD3^−^CD56^+^ NK cells were increased in the patients with intron22 and R382Q mutation (Fig. 3l-n). Similar results were found in NKT cells. The proportion of CX3CR1^+^ NKT cells was increased in intron22 and R382Q mutation patients (Fig. 3o).

Previous studies have shown that CX3CR1^+^ NK cells represent a late stage of NK cells with reduced cytotoxicity [26]. The expression of CD57 is a terminally differentiated marker of NK cells [27]. Thus, we investigated the co-expression of CX3CR1 and CD57 in NK cell subsets. Gated from CD3^−^CD56^+^ NK cells, our results showed increased CX3CR1^+^CD57^+^ NK cells and decreased CX3CR1^+^CD57^−^ NK cells in the intron22 and R382Q mutation patients (Fig. 3g-h, p-q). Overall, the increased amount of terminally differentiated CX3CR1^+^CD57^+^ NK cells observed in our patients with intron22 and R382Q mutations suggests that terminal differentiation of NK cells may be a common feature in patients with AD-HIES caused by *STAT3* mutations. Furthermore, such alteration of CX3CR1^+^CD57^+^ NK cells provides a potential explanation for the recurrent infections experienced by these patients.

In the patient with intron22 mutation, scRNA-seq revealed elevated expression of TIM3 in CX3CR1^+^ cells, a marker indicative of NK cell maturation or exhaustion [23]. Other immune cell functional markers such as LAG3, KLRG1, and CD57 were also highly expressed in CX3CR1^+^ NK and NKT cells (Fig. S2e), suggesting a state of hyperactivation and functional exhaustion. Further experiments confirmed that the MFI of TIM3 was elevated in both NK and NKT cells, although more significantly elevated in CX3CR1^+^ NK cells than CX3CR1^−^ NK cells (Fig. S2f-g). These results suggest that the NK cells have reached a terminal differentiation stage with potential senescence in *STAT3* mutant samples. Additionally, PRF1 and GZMB are key killer molecules in cytotoxic lymphocytes such as NK and T cells [28]. A scRNA-seq analysis of CX3CR1^+^ NK and T cells showed a significant increase in the levels of PRF1 and GZMB (Fig. S2h), indicating a state of overactivation that could further induce immunosuppression. Next, we investigated the functional changes in immune cells of the patient with intron22 mutation by flow cytometry. Compared to the HC, CD56^dim^ NK cells exhibited an elevated level of apoptosis without a corresponding change in proliferation capacity (Fig. S2i), and NKT cells showed an increase in proliferation, yet their apoptosis levels remained unaffected (Fig. S2j). When we expanded our analysis to include T and B cells, we discovered that B and T cells showed an increase in apoptosis alongside a decrease in their proliferation rates (Fig. S2k-l). IL-10 is an important anti-inflammatory cytokine that inhibits inflammatory responses and regulates immune system homeostasis [29]. We found that the proportion of IL-10^+^ cells was significantly reduced in the intron22 mutation patient’s NK and NKT cells (Fig. 3r-s), suggesting an immune imbalance leading to excessive immune activation and immune responses.

These observations suggest that NK cells from patients with intron22 mutation are likely to be towards the terminal stage of differentiation, and that the *STAT3* mutation triggers a cascade of events leading to the hyperactivation of NK cells, which subsequently results in cellular exhaustion and an immunosuppressive state.

### STAT3 inhibits CX3CR1 expression by binding to the CX3CR1 promoter

**Fig. 4:**
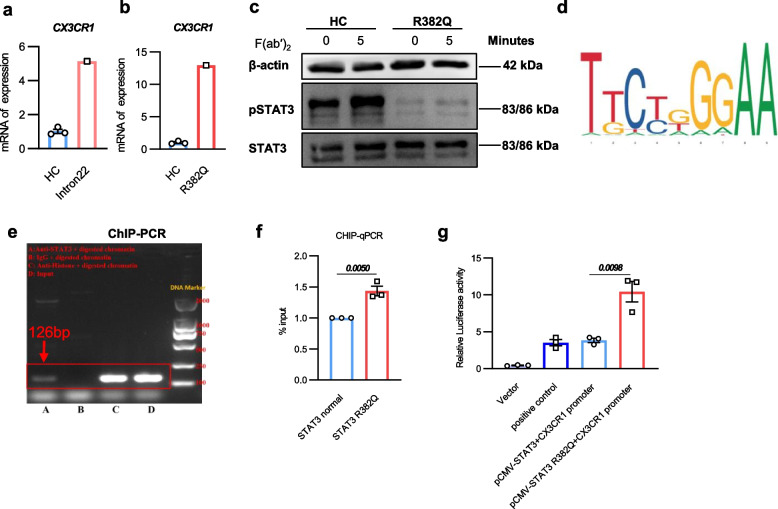
STAT3 inhibits CX3CR1 expression by binding to the *CX3CR1* promoter. **a**-**b** qPCR analysis of *CX3CR1* mRNA levels in PBMCs from patients with intron22 (**a**) and R382Q mutation (**b**), compared to three HCs. **c** Immunoblot analysis of total STAT3 and pSTAT3 levels in PBMCs from patient with R382Q mutation and HC. **d** Binding site prediction of STAT3 within the promoter region of *CX3CR1* gene, generated using the JASPAR database (http://jaspar.genereg.net). **e**–**f** ChIP assay analysis of STAT3 binding to the *CX3CR1* promoter in 293 T cells transfected with either wild-type STAT3 or R382Q mutant. Binding was assessed by PCR (**e**) and qPCR (**f**). **g** 293 T cells transfected with the pGL3-*CX3CR1* promoter together with pCMV-*STAT3* or pCMV-*STAT3 R382Q* and pRL-TK (internal control) were used for the luciferase reporter assay. Each experiment was independently repeated at least 3 times for Fig. 4e-g. Two-tailed unpaired *t* test, **P < 0.01

To explore the potential mechanism by which *STAT3* mutations lead to an increase in CX3CR1, we detected the mRNA levels of *CX3CR1* in PBMCs of the *STAT3* mutant patients by qPCR. The mRNA levels of *CX3CR1* in *STAT3* mutant patients were significantly higher than those of HCs (Fig. 4a-b). Additionally, in the patient with R382Q mutation, the levels of STAT3 protein were comparable, but the pSTAT3 was significantly reduced (Fig. 4c). Therefore, we further investigated whether STAT3 has regulatory roles on CX3CR1. By using JASPAR website [30], we predicted the possible binding site of STAT3 in the promoter of *CX3CR1*, which is TTTCAGGAA (Fig. 4d). To assess this, STAT3 was ectopically expressed in 293 T cells, then ChIP assays were conducted with an anti-STAT3 antibody to identify the promoter of *CX3CR1* that was evaluated by ChIP-PCR. Results showed that STAT3 does bind to the *CX3CR1* promoter (Fig. 4e). Then we overexpressed wild-type *STAT3* and *STAT3* R382Q mutants in 293 T cells and performed ChIP-qPCR. The results suggested that the *STAT3* R382Q mutation enhances its ability to bind the promoter region of *CX3CR1* (Fig. 4f). This could be due to conformational changes increasing the accessibility of the DNA-binding domain. Furthermore, we amplified the promoter region of the *CX3CR1* gene and conducted dual-luciferase reporter gene assays by co-transfecting the promoter-containing plasmid with a *STAT3* expression plasmid or a *STAT3* R382Q mutated plasmid into the 293 T cell line. The results were consistent with the ChIP-qPCR results, indicating that STAT3, as a transcription factor, indeed activates the transcriptional activity of the *CX3CR1* promoter, and mutated *STAT3* exhibits stronger regulatory activity of *CX3CR1* (Fig. 4g). Together, these findings demonstrate the association between STAT3 and CX3CR1 in our *STAT3* mutant patients, providing a mechanism for increased levels of CX3CR1^+^ NK and NKT cells.

### *STAT3* mutation disrupts T-cell differentiation and immune homeostasis

**Fig. 5:**
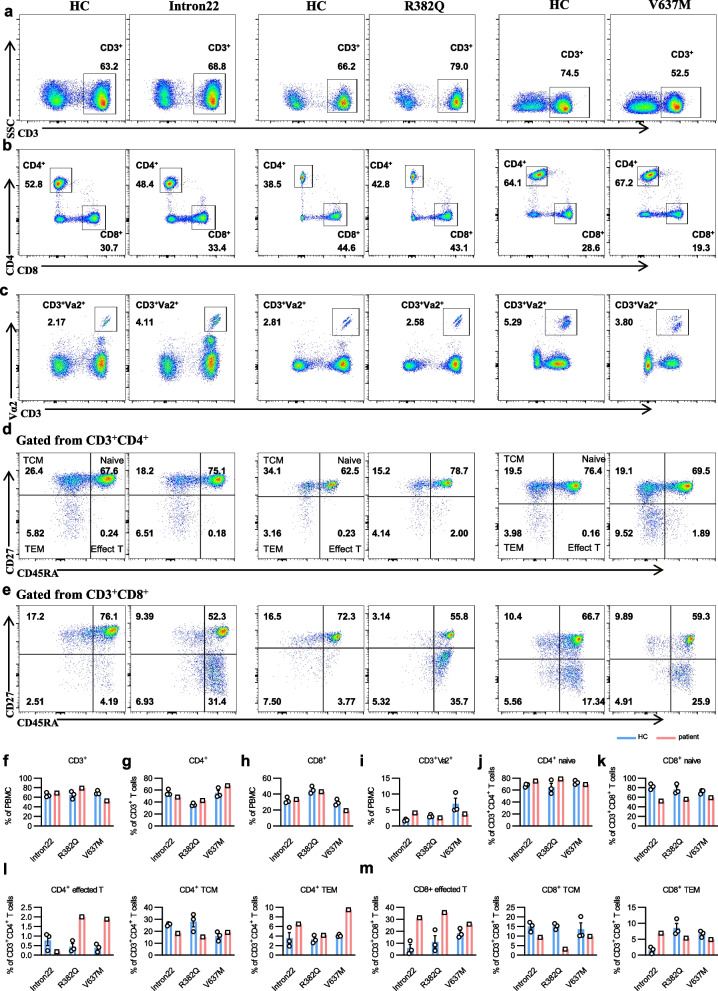
*STAT3* mutation leads to aberrant T-cell fate. **a**-**c** FCM analysis of CD3^+^, CD4^+^, CD8^+^, and CD3^+^Vα2^+^ T cells subsets from three HCs and *STAT3* mutant patients. Shown are representative dot plots. **d**-**e** FCM analysis of T cell subsets (TCM, naïve, TEM, and effector T cells) from CD3^+^CD4^+^ and CD3^+^CD8^+^ T cells. Shown are representative dot plots. **f**-**i** Statistics of percentage (± SEM) of CD3^+^, CD3^+^CD4^+^, CD3^+^CD8^+^, and CD3^+^Vα2^+^ T cells in PBMCs from three HCs and *STAT3* mutant patients. **j**-**m** Statistics of percentage (± SEM) of CD3^+^CD4^+^ naïve T, CD3^+^CD8^+^ naïve T cells, TCM, TEM, effector T cells in CD3^+^CD4^+^, and CD3^+^CD8^+^ T cells from three HCs and the *STAT3* mutant patients

STAT3 plays a crucial role in the differentiation and development of T cells [31]. To analyze the impact of *STAT3* mutations on T cell differentiation, we compared the proportions of CD3^+^, CD3^+^CD4^+^, CD3^+^Vα2^+^, and CD3^+^CD8^+^ T cells in PBMCs between *STAT3* mutation patients and HCs. Our analysis revealed no significant differences in T cell proportions for the intron22 and R382Q mutations. However, in the case of the V637M mutation, we observed a decrease in CD3^+^ and CD3^+^CD8^+^ T cells (Fig. 5a-c, f-i).

We further characterized memory and effector T cells using CD45RA and CD27 markers. In all three patients, the CD4^+^ naïve T cells (CD45RA^+^CD27^+^) had no significant changes (Fig. 5d, j). However, the CD3^+^CD4^+^ effector T cells (CD45RA^+^CD27^−^) were reduced in the intron22 mutation patient, while increased in patients with R382Q and V637M mutations. The CD3^+^CD4^+^ TEM were decreased or had no changes in the *STAT3* mutation patients (Fig. 5d, l). For CD3^+^CD8^+^ T cells, the naïve T cells and TCM proportions were reduced, while the proportions of effector T cells increased in all three patients (Fig. 5e, k, m). These findings indicate that *STAT3* mutation disrupts T-cell differentiation and immune homeostasis.

### Binding of STAT3 to *IL-4* promotes Th2 cell response, leading to elevated IgE

**Fig. 6:**
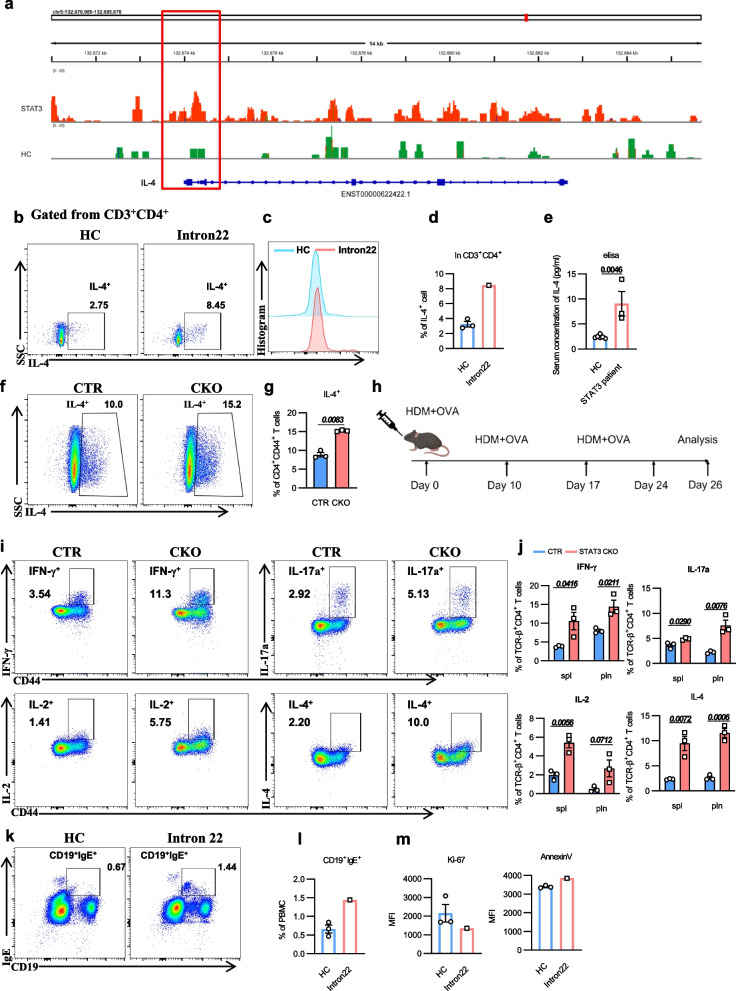
Binding of STAT3 to *IL-4* promoter promotes Th2 response, leading to elevated IgE. **a** The CUT&Tag analysis results in the transcription region of *IL-4* on CD4^+^ T cells from a HC and *STAT3* intron22 mutant patient. **b**-**c** FCM analysis of the IL-4^+^ T cells from three HCs and the *STAT3* intron22 mutant patient. Shown are representative dot plots. **d** Statistics of percentage (± SEM) of IL-4^+^ T cells from three HCs and *STAT3* intron22 mutant patient. **e** The level of IL-4 cytokine in serum was analyzed by ELISA. (HC = 6, patient = 3). **f**-**g** Sorted CD4^+^ T cells from STAT3 CKO mice were inducted to Th2 cell polarization with anti-CD3/28, IL-4, anti-IFN-γ and anti-IL-2 in vitro. Cells were collected five days later and followed by flow cytometry analysis. IL-4^+^ represents Th2 cells. Representative plots of flow cytometry. (*n* = 3). **h** Flow chart for the construction of a mouse allergy model using HDM and OVA sensitization. **i**-**j** Flow analysis of cytokine expression in STAT3 CKO and control mice sensitized with HDM and OVA. Representative plots of flow cytometry (**i**). The percentages of IFN-γ^+^, IL-17^+^, IL-2^+^, and IL-4^+^ were analyzed in CD4^+^ T cells (**j**). (*n* = 3). **k**-**l** Flow analysis of CD19^+^IgE^+^ B cells from three HCs and patient with the *STAT3* intron22 mutation. Shown are representative dot plots (**k**). The percentage (± SEM) of CD19^+^IgE^+^ B cells was shown in (**l**). **m** The MFI of Ki-67 and AnnexinV in CD19^+^IgE^+^ cells from three HCs and the *STAT3* intron22 mutant patient. Two-tailed unpaired *t* test, *P < 0.05, **P < 0.01, ***P < 0.001

T cell differentiation plays a crucial role in IgE production, with a particular emphasis on the involvement of Th2 cells [32]. We sorted CD4^+^ T cells from the PBMCs of the patient with intron22 mutation and HC, and performed chromatin occupancy profiling of STAT3 using CUT&Tag. The analysis results revealed significantly enhanced binding of STAT3 to the promoter region of the *IL-4* gene (Fig. 6a). This increased binding suggests a potential alteration in *IL-4* gene expression, which could subsequently affect the modulation of immune responses, particularly within the Th2-type immune response. Specifically, the enhanced recruitment of STAT3 near the TSS indicates a transcriptional regulatory mechanism through which STAT3 facilitates the transcriptional activation of *IL-4*, potentially playing a pivotal role in allergic reactions and immune regulation.

Furthermore, we assessed the levels of cytokines secreted by T cells. Elevated levels of IL-4 were detected in CD3^+^CD4^+^ T cells (Fig. 6b-d). Meanwhile, we also measured the level of IL-4 cytokine in the patients’ serum. Results showed that in the serum of patients with *STAT3* mutations, the levels of IL-4 cytokine were significantly higher than that of HC (Fig. 6e). Additionally, we isolated the CD4^+^ T cells from STAT3-CKO mice and induced Th2 polarization in vitro. The results showed that, under the same polarization induction conditions, the STAT3-deficient T cells differentiated more efficiently into the Th2 phenotype (Fig. 6f-g). Next, we established an allergy model by sensitizing STAT3-CKO and control mice with dust mites (HDM) and OVA (Fig. 6h). After two rounds of intensified sensitization, we detected cytokine secretion profiles from T cells. We found that while the levels of several cytokines were elevated in STAT3-deficient T cells, the increase in IL-4 production was the most prominent (Fig. 6i-j). The above results indicate that in the absence of STAT3, T cells exhibit a preferential skewing towards a Th2 differentiation pathway. This in vivo finding in the CKO mouse model is consistent with our observations in patients, where *STAT3* mutations are associated with enhanced binding of STAT3 to the *IL-4* TSS region, potentially contributing to the Th2 bias.

To determine whether this Th2 cell differentiation indeed caused elevated IgE levels, we examined the proportion of IgE^+^ cells in B cells from the patient with intron22 mutation. The proportion of CD19^+^IgE^+^ cells was significantly higher than in the control group (Fig. 6k-l). Additionally, in IgE^+^ B cells, the MFI of Ki-67 decreased, while the Annexin V increased (Fig. 6m), suggesting that IgE^+^ B cells were undergoing rapid apoptosis. Overall, our results indicate that *STAT3* mutations promote Th2 cell differentiation by increasing binding to the *IL-4* TSS region, which, in turn, leads to increased IgE production.

### Different mutation sites of *STAT3* have different effects on B cell differentiation

**Fig. 7:**
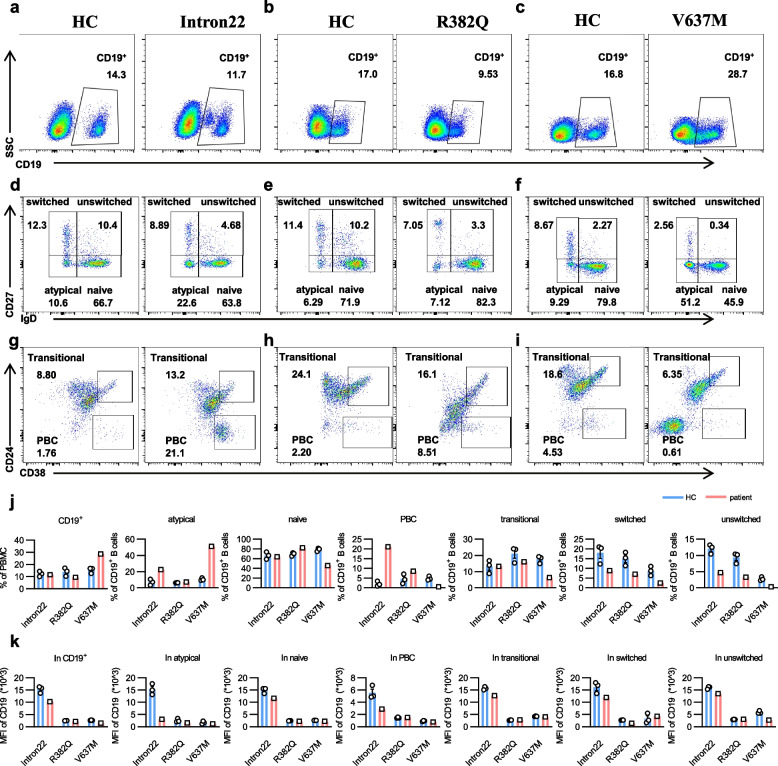
*STAT3* mutation affects B cell frequency and differentiation. **a**-**i** Flow analysis of CD19^+^ B cells, naive B, switched & unswitched B cells, atypical B cells, transitional B cells, and plasmablast cells in PBMCs from three HCs and patients with *STAT3* mutation. Shown are representative dot plots. **j** The percentage (± SEM) of those B cells shown in (**a**-**i**). **k** Analysis of the MFI of CD19 in B cell subsets from three HCs and *STAT3* mutant patients

Specific B cell subsets play pivotal roles in immune responses to infections, contributing to the generation of robust and targeted humoral immunity [33]. To investigate whether *STAT3* mutation affects the immunophenotype of B cells, we assessed changes in the distribution of B cell subsets in PBMCs from age-matched HCs and the *STAT3* mutation patients. Our findings indicated no significant differences in the proportion of CD19^+^ B cells in intron22 and R382Q mutation patients, however, in the V637M mutation patient, the proportion of CD19^+^ B cells was increased significantly (Fig. 7a-c, j). The proportion of atypical B cells (IgD^−^CD27^−^) was increased in intron22 and V637M mutation patients. The naïve B cells (IgD^+^CD27^−^) (Fig. 7d-f, j) was comparable between the intron22 mutation patient and HCs, but increased in the R382Q mutation patient and decreased in the V637M mutation patient. The transitional (CD38^+^CD24^+^) B cells had no significant changes in patients with intron22 and R382Q mutations, but decreased in the V637M mutation patient. However, there was a notable increase in the proportion of plasmablasts (PBC) (CD38^+^CD24^−^) (Fig. 7g-i, j) in intron22 and R382Q mutation patients and decreased in the V637M mutation patient. More consistent across the three *STAT3* mutation patients were the reduction in the proportion of the switched (IgD^−^CD27^+^) and unswitched (IgD^+^CD27^+^) B cells (Fig. 7d-f, j). Additionally, the MFI of CD19 was markedly decreased in various phenotypes of B cells in the *STAT3* mutation patients (Fig. 7k). Although different *STAT3* mutation sites have different effects on B-cell differentiation, *STAT3* mutations all affect the expression of CD19 on the surface of B cells. Here, we summarized a table of the effects of different *STAT3* mutation sites on cellular immune phenotypes to facilitate future clinical screening for children with *STAT3* mutations (Supplemental Table 1).

## Discussion

In this study, we report a case of HIES caused by a LOF mutation in intron22 of *STAT3* (2144 + 1G > A). scRNA-seq of this patient revealed a distinct population of CX3CR1^+^ NK and NKT cells. We further investigated the regulation of CX3CR1 by STAT3 in patients with R382Q and V637M mutations, demonstrating that STAT3 suppresses CX3CR1 expression by binding to its promoter. Moreover, CUT&Tag and flow cytometry analyses indicated that STAT3 deficiency leads to elevated IL-4 expression in CD4^+^ T cells, a potential mechanism underlying the observed immune dysregulation. Additionally, using a STAT3-CKO mouse model, we explored the broader impact of *STAT3* mutations on the homeostasis of various immune cell subsets, most prominently in regulating NK cell differentiation and T cell polarization.

By July 2020, 143 heterozygous STAT3 variants associated with HIES had been reported. These variants consist of 114 missense mutations (79%), 21 in-frame indels (15%), and 8 nonsense or frameshift indels (6%). *STAT3* has numerous mutation sites, with the majority being associated with dominant negative effects. Heterozygous germline mutations can result in LOF and GOF variants. LOF mutations have been associated with HIES, recurrent pulmonary infections, bone fragility, dermatitis, and mucocutaneous infections (cold abscesses), while GOF mutations can give rise to lymphoproliferation and autoimmune diseases. However, Lorenzo et al. reported a unique case of *STAT3* p.R335W (c.1003C > T) mutation, with HIES and Sjogren syndrome (SS). In that report, the authors concluded that with the many functions mediated by STAT3 and the heterogeneity of mutations, a simple GOF/LOF nomenclature does not cover the molecular complexity.

In our study, the *STAT3* intron22 (2144 + 1G > A) mutation patient had the main features of the so-called LOF type with HIES, including retained primary teeth and growth retardation. Moreover, the patient also exhibited an increased proportion of NKT and CX3CR1^+^ NK cells. The *STAT3* intron22 (2144 + 1G > A) mutation was first reported in 2010 with unknown effects at the protein level [34]. Later, Asano et al. examined this intron mutation by investigating *STAT3* variant A4 cells that were transfected with STAT3^−/−^ and found two transcripts named S701Kfs*17 (92%) and S701-D732delinsN (8%). They further revealed that both transcripts were nonfunctional, showing no activation after stimulation with IL-6 [11]. In our study, by using PBMCs from our pediatric patient, we found that the *STAT3* intron22 (2144 + 1G > A) mutation leads to reduced STAT3 phosphorylation compared to healthy controls, indicating impaired function of STAT3. Additionally, we found that *STAT3* mutations regulate the phenotype and function of immune cells by modulating CX3CR1 and IL-4, further elucidating the mechanisms of immune deficiency and high IgE levels in patients with *STAT3* mutations.

The CX3CL1/CX3CR1 chemokine/chemokine receptor pair plays a significant role in the immune response, particularly in the context of NK cell-mediated responses. Studies have shown that CX3CR1 is predominantly expressed on terminally differentiated NK cell subsets. Moreover, CD57, a marker of NK cell maturation, is expressed in conjunction with the loss of antigens typical of CD56^bright^ NK cells and the up-regulation of markers including CD16, KIR, and LIR-1 [35]. Frequencies of CD57-expressing cells in blood and tissues have been correlated with clinical prognosis in chronic infections or various cancers and with human aging [36]. TIM3 is recognized as a contributor to effector T cell exhaustion and is commonly found on the membranes of NK cells. Several studies suggested that TIM3 may serve as a marker of NK cell activation and maturation [37]. However, the precise dynamics of TIM3^+^ and TIM3^−^ NK cells remain inadequately characterized. In this study, our results revealed an increased frequency of CX3CR1^+^CD57^+^ NK cells in the patient with *STAT3* intron22 (2144 + 1G > A) mutation. This observation, in conjunction with the patient’s clinical manifestations of recurrent infections and weakened immunity, implies that CX3CR1^+^CD57^+^ NK cells are at a terminally differentiated stage.

Our scRNA-seq analysis, corroborated by subsequent experiments, revealed an elevated cytotoxic potential in CX3CR1^+^ NK and T cells. This finding implies a state of overactivation and a trend towards functional exhaustion. Additionally, few previous studies on *STAT3* mutations have mentioned the changes in the number of NKT cell subsets under mutated conditions. Our results revealed an elevated amount of CX3CR1^+^ NKT cells as well. NK and NKT cells serve as crucial components of the innate immune system, acting as the first line of defense against pathogens and tumor cells. The cytotoxicity of NK and NKT cells is essential for eliminating abnormal cells and maintaining immune surveillance, the quantitative and functional alterations observed in these cells provide an explanation for the immunodeficiency associated with *STAT3* mutations. In summary, we found that STAT3 influences the differentiation and function of NK and NKT cells by modulating CX3CR1, thereby providing an explanation for the immune deficiencies observed in patients with *STAT3* mutations.

Though the trend in T cell quantity changes is not completely consistent between *STAT3* intronic and exonic mutations, our results found that the *STAT3* mutations both primarily affect effector T and memory T cells. We have summarized the changes in immune cell phenotypes caused by different *STAT3* mutation sites in Supplemental Table 1. Meanwhile, mutations in *STAT3* intron22 (2144 + 1G > A) were found to promote Th2 differentiation in T cells. Our CUT&Tag results revealed that the binding of STAT3 to the TSS region of *IL-4* is enhanced in *STAT3* intron22 (2144 + 1G > A) mutation. This finding provides a potential mechanism between *STAT3* mutations and the trend toward Th2 differentiation, ultimately leading to elevated levels of IgE. Our findings further explore the mechanisms by which STAT3 contributes to HIES and propose that the TSS region could serve as a promising target for subsequent research.

This study has several limitations. First, due to the rarity of patients with *STAT3* mutations and the difficulty in obtaining clinical samples, our study was conducted on a small number of cases. Consequently, our conclusions, particularly those regarding the novel cell populations, require further validation in larger patient cohorts. Second, the direct correlation between the observed CX3CR1^+^CD57^+^ NK cell population and the clinical prognosis of HIES patients could not be established and warrants future investigation as more clinical data become available. Furthermore, our efforts to delve deeper into the underlying mechanisms were met with significant technical challenges. Attempts to generate mouse models with the *STAT3* R382Q or V637M point mutations resulted in embryonic lethality, with even heterozygous mice failing to survive. Similarly, technical constraints prevented the successful establishment of cell lines or animal models for the *STAT3* intron22 (2144 + 1G > A) mutation. Consequently, our study was largely restricted to phenotypic analyses of patient-derived PBMCs. Therefore, the precise molecular mechanisms by which STAT3 regulates CX3CR1 and the full clinical implications of this axis remain important areas for future exploration.

Overall, our study revealed an increase in CX3CR1^+^CD57^+^ NK and NKT cells in patients with *STAT3* mutation and identified the regulatory role of STAT3 in CX3CR1 expression, which explains the immune deficiency observed in these patients. Additionally, we analyzed changes in T cell phenotypes in patients with *STAT3* mutations and discovered that STAT3 promotes Th2 differentiation by enhancing *IL-4* expression, ultimately leading to increased levels of serum IgE. Taken together, our research enriches the clinical manifestations reported for the *STAT3* intron22 (2144 + 1G > A) mutation and explores the mechanisms by which *STAT3* mutations lead to immunodeficiency and HIES. The alterations in lymphocyte phenotypes have also been summarized for further exploration.

## Materials and methods

### Patients and control subjects

Patients with HIES were enrolled according to the following criteria. Inclusion criteria: (1) clinical diagnosis of HIES based on either an NIH HIES score ≥ 40 or a confirmed pathogenic STAT3 variant;

(2) serum total IgE ≥ 2000 IU/mL on two separate occasions; (3) documented history of recurrent skin and/or pulmonary infections. Exclusion criteria: (1) secondary causes of elevated IgE (e.g., parasitic infection, uncontrolled atopic dermatitis, and HIV); (2) other primary immunodeficiencies that could confound the immunophenotype;(3) systemic immunosuppressive or biological therapy within the previous 3 months;(4) inability to obtain informed consent. The clinical trial registration number is 2020GCP0304.

### Mice

*Vav*^icre^*Stat3*^fl/fl^ mice were obtained from Xiaohuan Guo (Tsinghua University, Beijing). All mice were bred in specific-pathogen-free conditions. Animal protocols were approved by the institutional animal care and use committee of Tongji Hospital, Tongji Medical College, Huazhong University of Science and Technology.

### Isolation of PBMCs

For obtaining human serum and PBMCs, peripheral blood collected in EDTA tubes was centrifuged at 3000 rpm for 10 min and then 2 mL of serum was removed and frozen. The rest of the sample was mixed with PBS and added to a centrifuge tube containing Ficoll‐Hypaque solution, which was then centrifuged with a gradual acceleration to 2000 rpm and a slow deceleration at the end.

### Whole genome sequencing

The genomic DNA of the submitted samples was extracted, fragmented, ligated, amplified, and purified, and then a DNA library was prepared by hybridization capture method, and then the exonic regions and the flanking intronic regions (20 bp) of 20,099 genes in the human whole exome were detected by high-throughput sequencing platform. The sequencing data were aligned with the reference sequence of human genome hg19 (GRCh37), and the coverage and sequencing quality of the target regions were evaluated.

### Single-cell RNA sequencing and analysis

Seurat (v5.0.1) was used for gene expression analysis. Cells were projected onto a UMAP plot based on gene expression similarity.  Canonical gene markers were used to annotate PBMC’s clusters (detailed in table S1). The FindMarkers function was used to calculate DEGs of two different cell subsets, and Wilcoxon rank-sum test was used to calculate p values and fold change (FC) values. The gseGO function in clusterProfiler (v4.10.0) was used to perform GSEA on the gene ontology of the DEGs, and the gseKEGG function in clusterProfiler (v4.10.0) was used to perform GSEA on the KEGG. The monocle (v2.30.0) and CellChat (v1.5.0) R package were used to construct single cell pseudotime trajectories and analyze cell-cell interaction, respectively.

### CUT&Tag approach and analysis

CD4^+^ T cells were incubated with ConA Beads Pro at 25°C for 10 min, anti-STAT3 antibody was added and incubated at 4°C overnight. Next was incubation with secondary antibodies at room temperature. Then the samples were incubated and fragmented with Mgcl2. The DNA was extracted and amplified using indexing primers. Bcl2fastq v2.20 with fastqc was used to de-multiplexed the raw data for quality control. Clean reads were mapped to reference genome GRh38 by Bowtie2. Reads were mapped to E. coli genome DH10B for Spike-in mapping. Duplicated reads were removed, while uniquely mapped reads were kept. Spike-in normalization was achieved through multiply primary genome coverage by scale factor (100000/fragments mapped to E. coli genome). CUT&Tag peaks were called by SECAR. Track visualization was done by bedGraphToBigWig, bigwig files were imported to Integrative Genomics Viewer for visualization.

### Flow cytometry (FCM)

PBMCs from HCs and mutant patients were stained with the antibodies in supplemental Table 3 for 30 min at 4°C refrigerator, then washed twice. Flow cytometric data were acquired with an Attune NxT (Thermo Fisher) and analyzed with FlowJo 10 software (TreeStar, Ashland, Ore).

### Cytokine assay

Single-cell suspensions of PBMCs were stimulated with PMA and Ionomycin at 37C° for 5 h. Then labelled with anti-CD3 APC-Cy7, anti-CD4 PE, anti-CD56 Pacific Blue, washed twice with 2% PBS, then fixed, permeabilized and labelled with anti-IL-4 BV421, anti-IL-10 PE.

### Western blot

PBMCs (2.5 × 10^6^) were stimulated with 5 μg/mL Biotin-F(ab’)_2_ anti-human Ig(M + G) for 30 min and then incubated for 5 min at 37°C and lysed by RIPA buffer. Proteins were separated using 8% SDS-PAGE and then incubated with anti-STAT3 antibody (Cat#A22264, CST) and anti-Phospho-STAT3 antibody (Cat#9145S, CST).

### Th2 cell induction in vitro

Purified CD4^+^ T cells were seeded at a density of 2 × 10^5^ cells per well in 96-well plates pre-coated with anti-CD3. Cells were cultured in 200 µL of complete medium supplemented with soluble anti-CD28 mAb (1 µg/mL), recombinant mouse IL-4 (20 ng/mL), rmIL-2 (10 ng/mL), and an anti-IFN-γ neutralizing antibody (5 µg/mL). The cells were incubated at 37°C for 5 d. After 5 days of culture, cells were collected and stimulated with PMA, Ionomycin, and GolgiStop at 37°C for five hours followed by staining for IL-4, IFN-γ, IL-2, and IL-17A. IL-4^+^ was used to identify Th2 cells.

### Real-time PCR

Total RNA from PBMCs (1 × 10^6^) were isolated using the classical phenol-chloroform extraction. And then retro-transcribed to cDNA using an RT Reagent Kit from Abclonal, and analyzed for cDNA expression levels by 2 × Universal SYBR green fast qPCR mix using a Real Time PCR system. Finally, the Ct value was analyzed for gene expression by the 2^−ΔΔct^ formula with the primer sequences. Primer sequences were as follows: *SOCS3* forward primer CCTGCGCCTCAAGACCTTC and reverse primer GTCACTGCGCTCCAGTAGAA; *STAT3* forward primer CAGCAGCTTGACACACGGTA and reverse primer AAACACCAAAGTGGCATGTGA; *GAPDH* forward primer ACCCAGAAGACTGTGGATGG and reverse primer TTCTAGACGGCAGGTCAGGT.

### Cell culture, transfection and dual-luciferase reporter assay

The 293 T cell line used in this study was purchased from ATCC and identified by short tandem repeat (STR) analysis. It was confirmed to be free of mycoplasma contamination prior to the experiment. 293 T cells were cultured in DMEM containing 1% penicillin–streptomycin and 10% fetal bovine serum. The cells were grown to the fourth generation and then plated in a 24-well plate. When the cells reached 80% confluency they were transfected with pRL-TK and the target plasmid (pGL3-CX3CR1 promoter and pCMV-STAT3 or pCMV-STAT3 R382Q mutant plasmid) using vazyme’s transfection reagent. After culturing at 37°C for 48 h, the cells were collected and lysed for luciferase detection. Firefly luciferase activity was normalized to Renilla luciferase activity, and relative luciferase activity was calculated as the ratio of the experimental group to the control group. Each experiment was performed in triplicate and repeated independently at least three times.

### ChIP assay and ChIP-PCR

Chromatin immunoprecipitation (ChIP) studies were performed by the manufacturer’s protocol. 293 T cells overexpressing the STAT3 and *STAT3* R382Q mutants were treated with 1% formaldehyde at room temperature for 20 min, and terminated with 1.375 M of glycine. Then cytoplasmic lysate and nuclear lysate were added sequentially, collecting the supernatant for sonication to fragment the cross-linked chromatin, then immunoprecipitated with antibody against STAT3 and PCR measurements were performed on a qTOWER3/G PCR instrument. The primers for homo sapiens *CX3CR1* promoter were as follows: forward TCGAGTGCAGCCTGCAGAAG and reverse TGGTGAAGGCCTGGATCAGAAAG.

### Enzyme-linked immunosorbent assay (ELISA)

Serum levels of IL-4 and CX3CR1 were measured using commercially available ELISA kits (Human IL-4 ELISA kit, Cat# EHC006.96; Human CX3CR1 ELISA Kit, Cat# EH1786) according to the manufacturer’s instructions. All samples and standards were assayed in duplicate. Absorbance was measured at 450 nm using a microplate reader, and concentrations were calculated based on the standard curve generated from serial dilutions of the reference standard.

### Antibodies and reagents

Antibodies and reagents used in this study are listed in Supplemental Table 3.

### Statistical analysis

The normality of all the data was examined and two‐tailed unpaired *t*‐test was used (Prism 9.5, GraphPad Software). Data were extracted from at least three individual experiments. The figures shown are representative figures. Error bars were shown as mean (± SEM). *p* < 0.05 represented significant difference.

## Supplementary Information

Supplementary Material 1.

## Data Availability

The authors declare that all relevant data are available upon reasonable request. The scRNA-seq data for healthy controls has been deposited in the Gene Expression Omnibus (https://www.ncbi.nlm.nih.gov/geo/) under accession number GSE243002 and the scRNA-seq data for STAT3 mutant patient has been deposited in the Genome Sequence Archive for Human (https://ngdc.cncb.ac.cn/gsa) in the National Genomics Data Center (NGDC), China National Center for Bioinformation (CNCB), under accession number HRA012961.
