# Supplementary material for: STAT3 regulates NK and NKT cell differentiation through C-X3-C motif chemokine receptor 1 (CX3CR1) in hyper-IgE syndrome
Source: Mol Biomed. 2025 Nov 10;6:104. doi: 10.1186/s43556-025-00323-1 (PMC12602802; doi:10.1186/s43556-025-00323-1)
Supplement: Supplementary file 1 — Supplementary Material 1. [file 43556_2025_323_MOESM1_ESM.docx]

***STAT3* Regulates NK and NKT Cell Differentiation through C-X3-C motif Chemokine Receptor 1 (CX3CR1) in Hyper-IgE Syndrome**

Ju Liu^1^, Jingzhi Yang^3^, Jianing Tang^1^, Hongxia Tang^4^, Xin Dai^6^, Peiyao Jin^7^, Yanmei Huang^1^, Zhenzhen Li^1^, Ziyin Zhang^1^, Xiaohuan Guo^5^, Martin Bitzan^8^, Xiaoling Yin^2*^, Chaohong Liu^1*^

**Supplementary Information**

**Figure S1. Pseudo-temporal analysis reveals hyperactivated immune cells in children with *STAT3* mutations**

**
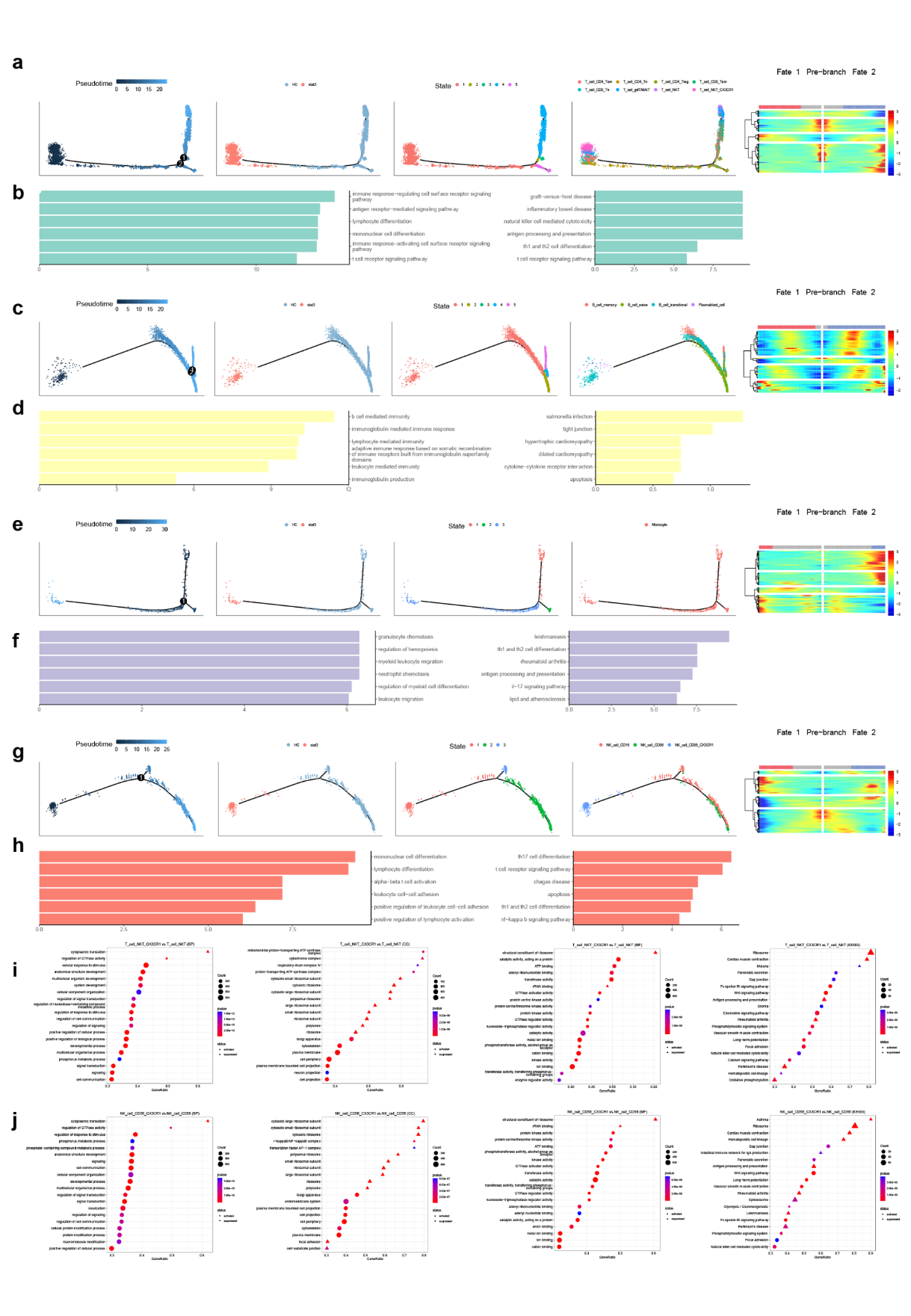
**

(a-g) Pseudo-time analysis results of T cells (a), B cells (c), monocytes (e) and NK cells (g) by pseudo-time, status, cell state, cell subtype, and heatmap of DEGs on both sides of branch point 1. And the GSEA results of changed genes based on branch expression of T cells (b), B cells (d), monocytes (f) and NK cells (h).

(i-j) GSEA results of differential expression genes (DEGs) between CX3CR1^+^ NKT cells and NKT cells (i), and CX3CR1^+^ NK cells and NK cells (j) on BP, MF, CC, and KEGG.

**Figure S2. *STAT3* mutations affected NK cell differentiation and lead to hyperactivation of NK cells, causing cellular exhaustion.**

**
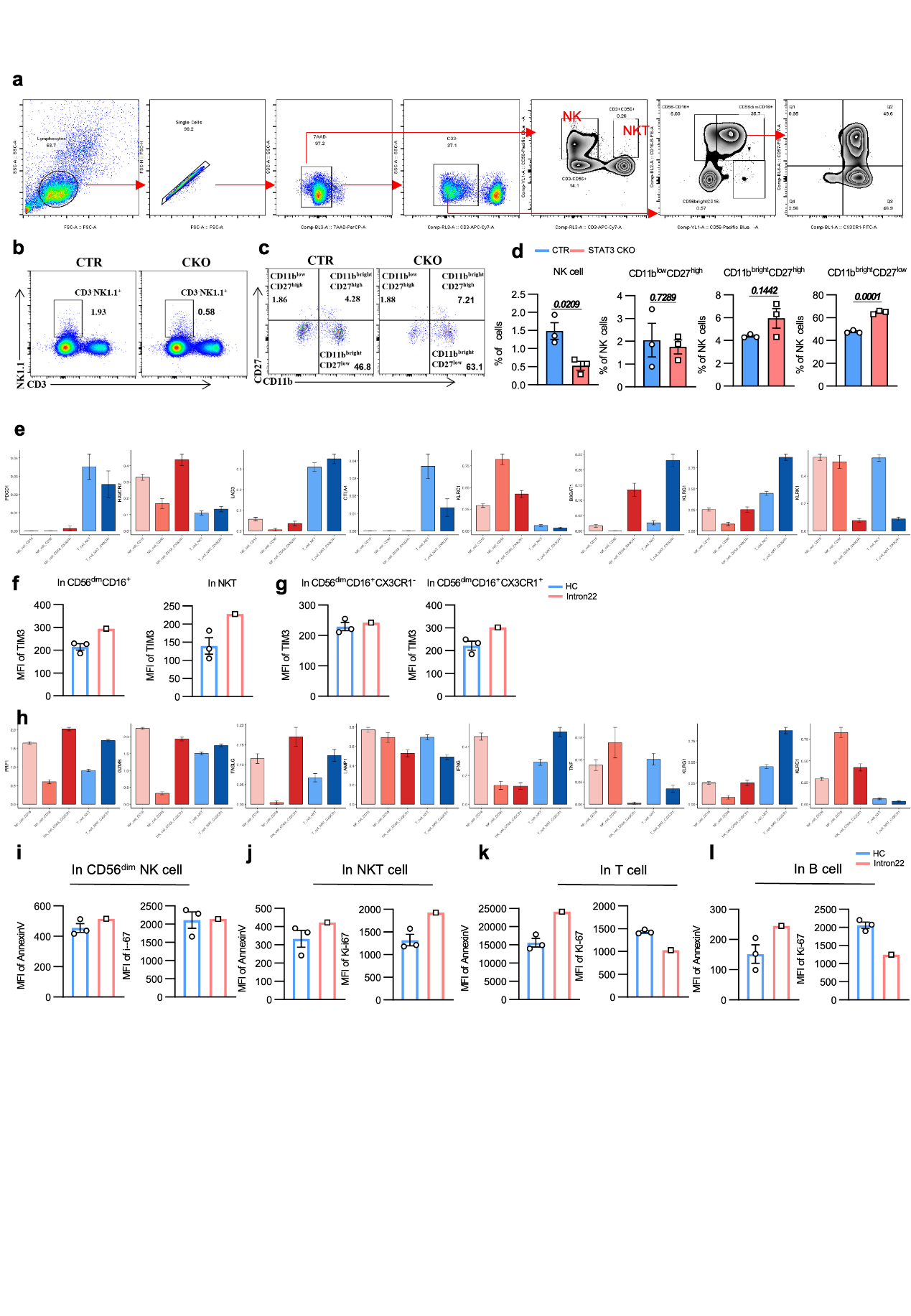
**

(a) The gate strategy of NK and NKT cells, CX3CR1^+^CD57^+^ NK cells.

(b-d) Flow analysis of NK subpopulation in STAT3 CKO mice. Shown are representative dot plots (b). The percentages of NK(CD3^-^NK1.1^+^) cells, NK subpopulation (CD11b^low^CD27^high^, CD11b^bright^CD27^high^, CD11b^bright^CD27^low^) were analyzed (d). (n = 3)

(e) The expression level of PD-1 (PDCD1), TIM3 (HAVCR2), LAG3, CTLA4, NKG2A (KLRC1), KLRG1, and CD57 (B3GTA1) on NK cells and NKT cells from scRNA-seq data.

(f-g) The MFI of TIM3 in CD56^dim^CD16^+^ NK and NKT cells (f), and in CD56^dim^CD16^+^CX3CR1^+^ or CD56^dim^CD16^+^CX3CR1^-^ cells (g) from three HCs and the *STAT3* intron22 mutant patient was analyzed by FCM.

(h) The expression level of PRF1, GZMB, FasL (FASLG), CD107A (LAMP1), IFN-γ (IFNG), TNF-α (TNF), and NKG2D (KLRK1) on NK and NKT cells from scRNA-seq data.

(i-l) Flow analysis proliferation and apoptosis of NK cells (i), NKT cells (j), T cells (k), B cells(l). (HC=3)

**Supplemental Table 1: Summary of immunophenotypes of patients with different mutation sites in *STAT3.***

| Patient ID | Patient1 | Patient2 | Patient3 |
| --- | --- | --- | --- |
| Sex | Male | Male | Female |
| Mutation Site | Intron22(2144+1G>A) | R382Q | V637M |
| Age at sample collection | 7 years and 3 months | 2 years and 8 months | 12 years and 5 months |
| B cell subsets | | | |
| CD19^+^ | - | - | up |
| Naïve | - | - | down |
| Atypical | up | - | up |
| Switched | down | down | down |
| unswitched | down | down | down |
| Transitional | - | - | down |
| PBC | up | up | down |
| T cell subsets | | | |
| CD3^+^ | - | - | down |
| CD3^+^Vα2^+^ | up | down | down |
| CD3^+^CD4^+^ | - | - | - |
| CD4^+^ naïve | - | - | - |
| CD4^+^ effect T | down | up | up |
| CD4^+^ TEM | - | down | up |
| CD4^+^ TCM | - | - | up |
| CD3^+^CD8^+^ | - | - | down |
| CD8^+^ naïve | down | - | - |
| CD8^+^ effect T | up | up | up |
| CD8^+^ TEM | up | down | - |
| CD8^+^ TCM | down | down | down |
| NK & NKT cell subsets | | | |
| CD3^-^CD56^+^ | up | down | down |
| CD56^-^CD16^+^ | - | down | down |
| CD56^dim^CD16^+^ | up | down | down |
| CD56^bright^CD16^-^ | down | down | down |
| NKT cell (CD3^+^CD56^+^) | up | up | up |

**Supplemental Table 2: Summary of cell cluster detailed.**

| Cell type | Marker |
| --- | --- |
| **B cell** | MS4A1^+^CD79A^+^ |
| transitional B cell | CD27^-^CD38^+^ |
| naïve B cell | CD27^-^CD38^-^ |
| memory B cell | CD27^+^CD38^-^ |
| Plasmablast cell | MZB1^+^CD79A^+^ |
| monocyt | LYZ^+^ |
| **Natural Killer (NK)** | KLRF1^+^ |
| CD16^+^ NK cell | FCGR3A^+^NCAM1^-^ |
| CD56^+^ NK cell | NCAM1^+^FCGR3A^-^ |
| CD56^+^CX3CR1^+^ NK cell | FCGR3A^+^NCAM1^+^CX3CR1^+^ |
| **T cell** | CD3D^+^ |
| CD4^+^ naïve T cell (CD4^+^ Tn) | CD4^+^CCR7^+^ |
| CD8^+^ naïve T cell (CD8^+^ Tn) | CD8A^+^CCR7^+^ |
| CD4^+^ effector memory T cell (CD4^+^ TEM) | CD4^+^CCR7^-^S100A4^+^ |
| CD8^+^ effector memory T cell (CD8^+^ TEM) | CD8A^+^CCR7^-^S100A4^+^ |
| **NKT cell** | KLRG1^+^NKG7^+^FCGR3A^+^ |
| CX3CR1^+^ NKT cell | KLRG1^+^NKG7^+^FCGR3A^+^CX3CR1^+^ |
| mucosal-associated invariant T (MAIT) cell and γδ T cell | SLC4A10^+^TRAV1-2^+^ and TRGV9^+^TRDV2^+^ |
| plasmacytoid dendritic cell (pDCs) | LILRA4^+^ |
| platelets | PPBP^+^ |

**Supplemental Table 3: Antibodies and reagents**

| REAGENT or RESOURCE | SOURCE | IDENTIFIER |
| --- | --- | --- |
| **Antibodies for B cells** | | |
| anti-CD19 FITC | Biolegend | Cat#302206 |
| anti-CD24 PE | Biolegend | Cat#311106 |
| anti-CD27 APC | Biolegend | Cat#302810 |
| anti-CD38 Pacific Blue | Biolegend | Cat#356628 |
| anti-IgE APC-Cy7 | Biolegend | Cat# 325520 |
| anti-IgD BV510 | Biolegend | Cat#348220 |
| anti-CD19 Percp-Cy5.5 | Biolegend | Cat#302230 |
| anti-AnnexinV BV605 | BD Bioscience | Cat# 563974 |
| anti-Ki67 PE-Cy7 | Invitrogen | Cat# 25-5698-82 |
| 7-Amino-Actinomycin D | BD Bioscience | Cat# 559925 |
| **Antibodies for T cells** | | |
| anti-CD8 FITC | BioLegend | Cat#555366 |
| anti-CD4 PE | BioLegend | Cat#357404 |
| anti-CD4 APC | BD Bioscience | Cat#551980 |
| anti-CD3 APC-Cy7 | BioLegend | Cat#344818 |
| anti-CCR7 PE-Cy7 | BioLegend | Cat#353226 |
| anti-CD45RA BV605 | BioLegend | Cat#304134 |
| anti-Vδ2 PE-Cy7 | BioLegend | Cat#331422 |
| anti-IL-4 BV421 | BioLegend | Cat#500826 |
| **Antibodies for NK and NKT cells** | | |
| anti-CX3CR1 FITC | Biolegend | Cat#341605 |
| anti-CD16 PE | BD Bioscience | Cat#555407 |
| anti-Tim-3 PE-Cy7 | Biolegend | Cat#345013 |
| anti-CD56 Pacific Blue | Biolegend | Cat# 362520 |
| anti-CD3 APC-Cy7 | BioLegend | Cat#344818 |
| anti-CD57 PE-Cy7 | Biolegend | Cat#359623 |
| Anti-IL-10 PE | Biolegend | Cat#501403 |
| **Chemicals, peptides, and recombinant proteins** | | |
| PMA | Sigma-Aldrich | P1585 |
| Ionomycin | CST | 9995S |
| Golgi stop | BD Bioscience | 554724 |
| CD3/CD28 Dynabeads | Thermo Fisher | 11131D |
| F(ab’)_2_ anti-human Ig (M +G) | Jackson ImmunoResearch | 109-066-127 |
